# Efficient Speech Detection in Environmental Audio Using Acoustic Recognition and Knowledge Distillation

**DOI:** 10.3390/s24072046

**Published:** 2024-03-22

**Authors:** Drew Priebe, Burooj Ghani, Dan Stowell

**Affiliations:** 1 Department of Cognitive Science and Artificial Intelligence, Tilburg University, 5037 Tilburg, The Netherlands; 2 Naturalis Biodiversity Center, 2333 Leiden, The Netherlands; burooj.ghani@naturalis.nl

**Keywords:** passive acoustic monitoring, eco-acoustics, deep learning, knowledge distillation, bioacoustics, classification, transfer learning, speech detection

## Abstract

The ongoing biodiversity crisis, driven by factors such as land-use change and global warming, emphasizes the need for effective ecological monitoring methods. Acoustic monitoring of biodiversity has emerged as an important monitoring tool. Detecting human voices in soundscape monitoring projects is useful both for analyzing human disturbance and for privacy filtering. Despite significant strides in deep learning in recent years, the deployment of large neural networks on compact devices poses challenges due to memory and latency constraints. Our approach focuses on leveraging knowledge distillation techniques to design efficient, lightweight student models for speech detection in bioacoustics. In particular, we employed the MobileNetV3-Small-Pi model to create compact yet effective student architectures to compare against the larger EcoVAD teacher model, a well-regarded voice detection architecture in eco-acoustic monitoring. The comparative analysis included examining various configurations of the MobileNetV3-Small-Pi-derived student models to identify optimal performance. Additionally, a thorough evaluation of different distillation techniques was conducted to ascertain the most effective method for model selection. Our findings revealed that the distilled models exhibited comparable performance to the EcoVAD teacher model, indicating a promising approach to overcoming computational barriers for real-time ecological monitoring.

## 1. Introduction

Bioacoustics is the scientific discipline that focuses on sounds generated by animals [1]. The field offers insight into the behaviors, communication, and migration patterns of different species. Recent advances in computational bioacoustics, such as data storage and digital recording costs, have enabled the application of more advanced analytical approaches like deep learning [1]. While early deep learning methods focused on neural networks such as the multilayer perceptron (MLP), Convolutional Neural Network (CNN) and Recurrent Neural Network (RNN) models currently surpass and exceed MLP models in the field [1]. More recently, a convolution-free Audio Spectrogram Transformer (AST), an attention-based model for audio classification, was designed [2]. However, due to the quadratic complexity of self-attention, transformer-based models such as AST are known to be computationally expensive, resulting in increased latency and model size when compared with lightweight CNNs [3].

Despite the recent progress in computational bioacoustics, some practical and theoretical obstacles remain that prevent deep learning methods from broad usage in the field. A notable obstacle arises from the intricacies of dealing with human speech recordings in wildlife settings. Although these recordings serve as a useful proxy for quantifying human disturbance in ecosystems, they also allow for a more precise assessment of human presence [4]. This increased precision, while beneficial in one aspect, could lead to significant data privacy concerns as acoustic monitoring equipment becomes more advanced and more widely implemented [1]. The implications of this obstacle extend even further given the documented impact of human activity on the temporal dynamics of animal activity patterns, which include an increase in nocturnality and potential consequences for ecological interactions [5,6,7]. Moreover, noise pollution levels in protected areas have doubled, affecting critical habitat areas for endangered species [8]. In response to these challenges, Cretois, Rosten, and Sethi [4] developed a voice activity detection (VAD) model, EcoVAD, aimed at addressing both the need for precise measurement of human presence and privacy preservation in eco-acoustic data.

In addition to the above-mentioned theoretical challenges, there are practical challenges that prevent deep models, such as EcoVAD, from being deployed in eco-acoustic environments. The deployment of such models has high latency costs [1]. The current state-of-the-art acoustic monitoring tool, AudioMoth [9], is a low-cost, low-power solution to certain technical challenges in bioacoustics. However, AudioMoth is not efficient enough to execute deep neural networks (DNNs) in real time [10]. The challenges that DNNs bring for deploying models on small devices have led to a series of model compression and acceleration techniques, one of which is knowledge distillation [11]. The main idea behind knowledge distillation is that a student model is trained to emulate the processing performed by a larger teacher model in order to distill refined knowledge and obtain a competitive performance versus the teacher [11]. This technique allows efficient DNNs to be trained from large DNNs without a substantial drop in accuracy [11].

While knowledge distillation addresses the compression framework required for deployment on edge devices, architectural efficiency remains another critical aspect for real-time inference [1,12]. In an attempt to design a more efficient architecture, Howard et al. [13] introduced the MobileNetV1 architecture, which replaced the convolutional layer of CNNs with depth-wise separable convolutions. Specifically, the utilization of factorized convolutions through the combination of depth-wise and point-wise convolution reduced the computation required by the convolutional block by a factor of eight [13]. While the introduction of MobileNetV1 allowed for a reduction in parameters without a significant loss in accuracy, it was not effective at efficiently extracting the manifold of interest (MOI) [14]. This issue was in part due to the application of the nonlinear functions (RELU) on low-dimensional activations, which lead to information loss in the MOI. To confront this problem within the MobileNetV1 architecture, ref. [14] introduced MobileNetV2, which incorporated inverted residuals with a linear bottleneck. In order to improve the representational power of the CNN architecture, Hu, Shen, and Sun [15] implemented a Squeeze-and-Excitation block (SE), which allows the weighting of interdependencies between channels for feature selection. In light of this development, researchers then attempted to augment MobileNetV2 and introduced the SE block in the MobileNetV3 architecture. As a result, this led to an improvement in both the latency and parameter size of the model [16].

Despite the design of MobileNet architectures addressing the model complexity and latency costs for deployment on small mobile devices, these architectures are not optimized for other edge devices, such as Raspberry Pi, NVIDIA Jetson Nano, or Google Coral, which contain different hardware specifications [17,18]. In an attempt to improve the MobileNetV3 design for Raspberry Pi devices, MobileNetV3-Small-Pi was developed [18]. This architecture replaced the 5x5 filter with a 3x3 filter in the convolution block and changed the hard-swish activation function to RELU. The modifications made to the MobileNetV3 led to improvements in both latency and accuracy in MobileNetV3-Small-Pi [18].

Silva et al. [19] built a CNN-based VAD model using audio spectrograms to detect speech in audio signals. Using the LeNet 5 CNN and the Half Total Error Rate metric, the proposed method outperformed several baseline VAD models in low-, medium-, and high-noise conditions. In an effort to further optimize VAD models in noisy conditions, ref. [20] integrated a two-layer bottleneck Denoising Autoencoder (DAE) with a CNN. The researchers carried out experiments using two different feature sets, MFCCs (Mel-Frequency Cepstral Coefficients) and filterbanks, and compared their performance in various Signal-to-Noise Ratio (SNR) conditions. The results demonstrate an improvement in classifying speech in high-noise environments. In an attempt to measure human disturbance in ecological settings, ref. [4] proposed an alternative approach for acoustic VAD models. Researchers trained CNN models on synthetic datasets containing human voices mixed with typical background noises encountered in eco-acoustic data. By proposing a specialized preprocessing pipeline for audio augmentation and synthetic dataset building, the results indicate the performance of a custom VGG11 model established a new state-of-the-art benchmark for VAD models in ecological settings. Despite the advances demonstrated in the aforementioned studies with respect to the accuracy of VAD models, the challenge of designing models that are suitable for real-time inference and deployment on edge devices remains a significant challenge. Ref. [21] proposed a lightweight CNN with data augmentation and regularization techniques to improve the generalization ability of the model. Utilizing the PreAct ResNet-18 architecture as a teacher and log-scaled Mel Spectrogram as feature inputs, researchers trained a student model using response-based distillation resulting in a lower equal error rate and latency from the distilled model. In a similar piece of research, ref. [22] proposed a response-based knowledge distillation approach, where the teacher estimates the frame probability for each sound event and provides frame-level supervision to the student model, which was trained to then discriminate ground truth speech from non-speech-labeled events. With the aim of deployment on embedded devices such as Raspberry Pi, the results indicate a 98% reduction in parameters while outperforming the teacher model.

This study addresses the challenge of deploying deep learning models for ecological speech detection within the computational constraints of small, edge devices. These cost-effective and low-power devices struggle to efficiently run complex neural networks like EcoVAD, hampering real-time bioacoustic monitoring. To circumvent these challenges, our research focuses on applying knowledge distillation to create streamlined student models that parallel the larger EcoVAD teacher model’s performance. This approach is intended to overcome the inherent memory, latency, and computational limitations of such devices while facilitating a more robust model capable of effective ecological monitoring.

## 2. Materials and Methods

In the current study, we build on the previous research discussed above, which has proven instrumental in developing efficient, compact deep learning models suitable for deployment. We design and execute experiments to optimize deep neural networks for real-time speech detection. To achieve this objective, we investigate the suitability of MobileNetV3-Small-Pi [18] model as a student architecture for EcoVAD [4]. The aforementioned studies also highlight the significance of specialized preprocessing, efficient lightweight architectures, and distillation techniques for optimizing VAD models for such deployment. Consequently, we employ different knowledge distillation techniques while incorporating variations in the MobileNetV3-Small-Pi architecture to achieve optimal performance. Finally, we examine how reductions in parameters, floating-point operations per second (FLOPs), multiplications, and memory utilization in student VAD models influence the performance of the resulting architectures.

### 2.1. Knowledge Distillation Techniques

Hinton, Vinyals, and Dean [23] first popularized the knowledge distillation method by training a smaller student network, using a teacher for distilled knowledge transfer. The method, known as response -based distillation, trains the student to optimize the loss function based on the student and teacher’s softened outputs. While response-based distillation allowed for “dark knowledge” to be distilled, depth is a critical aspect of feature representation learning [11,24].

In an attempt to distill intermediate representations, ref. [24] introduced feature-based distillation, which trained a student network to optimize the loss function based on the student’s outputs and ground truth labels, along with the feature maps from an intermediary layer within the student and teacher, respectively. This method, which selects a teacher hidden layer as a “hint” layer and student hidden layer as a “guide”, improved the generalization and accuracy of the student when compared with the teacher [11].

While featured distillation allowed for deeper representation learning, the knowledge distilled is independent of outside data examples. Thus, Park et al. [25] introduced relational knowledge distillation, a method relying upon the relations between learned representations. This method trained the student network to optimize the loss function based on the angle-wise and distance-wise relations between different data points, allowing the teacher to distill refined instance relations between the layers and outputs of the model [25].

### 2.2. Model Architectures

The teacher architecture used for knowledge distillation is based on a customized VGG11 architecture [4], adapted to process 128 × 128 single-color channel images, in contrast to the standard VGG11’s handling of 224 × 224 RGB images. Significant modifications included the reconfiguration of input and output neurons, the introduction of batch normalization after each convolutional layer, and the implementation of a dropout strategy in fully connected layers to enhance the model’s specificity for binary speech detection. Additionally, a Fast Fourier Transform (FFT) window duration of 64 milliseconds (equivalent to 1024 samples at a sampling rate of 16 kHz) with a 50% overlap (hop size of 512 samples) was selected for its proven effectiveness in audio classification tasks, as detailed in [4]. This approach is further validated by the findings of [26], particularly highlighting the significant role of normalizing the Mel Spectrograms along each frequency bin in enhancing classifier performance. By compressing the frequency into 128 Mel scale bands and implementing this normalization, the model’s input is finely tuned, thereby improving its capability to accurately differentiate between speech and nonspeech elements. All student architectures were based on MobileNetV3-Small-Pi (MSP) [18]. The student architectures maintained the differences implemented in [18] with respect to MobileNetV3, more specifically, the adjustment from a 5 × 5 filter with a 3 × 3 filter in the later convolution blocks and an adjustment from the hard-swish activation function to RELU. However, the architectural differences in the students differ from MSP in a number of ways.

With the goal of analyzing the efficiency of student architectures, four different student designs were trained to measure the tradeoffs in accuracy and efficiency. The primary differences between these four architectures lie within the number of channels used in the convolutional and bottleneck layers, as well as the overall depth of the architecture, allowing for an exploration of established principles [27] to find an optimal balance between model complexity and computational efficiency. Student 1 starts with an initial 3 × 3 convolutional layer with 16 output channels, followed by a series of bottleneck layers with channels ranging from 16 to 512. This design leverages concepts from residual learning to reduce the computational cost and enhance feature extraction capability compared with prior CNNs by using depth and channel expansion to capture complex patterns within the data [14]. Student 2, while similar to Student 1, has a reduction in the number of bottleneck layers and a difference in the input channels prior to the Adaptive Average Pooling layer. The input channels are changed from 256 to 512 in this case. The reduction in bottleneck layers allows for a continuation of the reduction in the depth of the network while maintaining a higher learning capacity for feature extraction in the later stages of the network.

Student 3 was initiated with a smaller number of channels compared with the aforementioned student architectures, starting at only 4 output channels in a 3 × 3 convolutional layer and progressing through a series of more compact bottleneck layers that scale from 4 to 128 channels. This architecture emphasizes an experimental divergence from its predecessors to examine efficiency with an inherent reduction in model capacity. The decrease in initial channels and compact bottleneck design was to ensure a reduction in calculations performed for each convolutional operation while ensuring computations performed within the bottleneck layers were reduced due to smaller feature maps. The final student, Student 4, maintains a similar foundational structure to Student 3, with a 3 × 3 convolutional layer with 4 output channels in the initial bottleneck. However, the number of bottleneck layers is reduced in this case, leading to a more compact architecture with fewer layers. The channel sizes range from 4 to 64. These design changes reflect our efforts to prioritize a reduction in depth and complexity in order to assess the generalization capabilities of a simplified network.

Each student architecture maintains a similar final layer construction, which consists of an Adaptive Average Pooling layer, two 1 × 1 convolutional layers, and a flatten layer. The models also maintain the presence or absence of the SE block, as in [18]. Furthermore, each student’s architecture maintains the same expansion ratio pattern, with the exception of Student 4. The differences in teacher and student architectures, which are highlighted in Table 1, influence each respective model’s capacity for feature extraction and performance on the voice activity detection task.

### 2.3. Dataset and Preprocessing

The current study used three distinct datasets for the EcoVAD preprocessing pipeline:

The Soundscape Dataset [4], collected from the Bymarka forest near Trondheim, Norway, contains a total of 10 days of acoustic data recorded in files of 55 s at a sampling frequency of 44.1 kHz. From the initial 10 days of recordings, a subset of data were used for the EcoVAD preprocessing pipeline, consisting of 9037 raw audio signals from a continuous 5-day forest recording sampled with the same rate and intervals.

The Libri-Speech Dataset [28], a corpus containing 1000 h of 16kHz of read English speech with a 1:1 male-to-female ratio was used for voice active detection. The data used for the EcoVAD preprocessing pipeline were a subset from the corpus containing 360 h, of which 200 h of English reading speech with a 1:1 male-to-female ratio was extracted.

The Background Noise Dataset is a combination of the ESC-50 dataset [29] and BirdClef 2017 dataset [30]. The ESC-50 dataset, used for environmental sound classification, contains 2000 environmental recordings organized in 50 classes. For training, we subsetted the data to only include 1600 recordings organized into 40 classes at 5 s intervals, removing human-related sounds. The BirdClef 2017 dataset, which includes audio recordings of various bird species, contains 36,496 audio recordings with 1500 species classes. Due to storage limitations, a subset of the dataset was used, accounting for 11,889 audio recordings belonging to 501 species. The three datasets, namely Soundscape, Libri-Speech, and Background Noise, were collectively utilized as inputs for the EcoVAD preprocessing pipeline.

The EcoVAD preprocessing pipeline [4] was used to generate a synthetic dataset consisting of 20,000 audio files, with a 1:1 distribution between speech and nonspeech audio files. The pipeline augments raw soundscape audio into processed 3 s soundscape audio clips, which are accompanied by ground truth labels denoting the presence or absence of speech. These processed 3 s soundscape audio clips were augmented with speech, background, and bird species audio recordings to build an accurate representation of the ecological soundscape. To refine the raw audio signals into features for the speech detection task, the signals were converted into 128 × 128 Mel Spectrograms containing a single color channel, as in [4]. The Mel Spectrograms were then used as input into the student and teacher architectures for training.

The Evaluation Playback Dataset [4] is an extensive collection of audio recordings designed to simulate diverse environmental conditions for the purpose of testing voice activity detection (VAD) models. This unique curated collection of three-second audio clips is derived from 48 two-minute recordings within forest and seminatural grassland environments. This dataset, consisting of 5140 audio files, incorporates audio recordings of male, female, and child voices, both in speech and nonspeech contexts, captured at distances of 1, 5, 10, and 20 m. The playback dataset allows for the final evaluation and verification of the robustness of the various student models across distinct landscapes and at varying distances.

### 2.4. Training and Evaluation

The synthetic dataset generated for training each student model was broken down into training, evaluation, and test sets with ratios of 60%, 20%, and 20%, respectively. All models utilized in this study were subjected to a training process that involved a maximum of 50 epochs, employing batch sizes of 32. The number of inputs for each model was set to the Mel Spectrograms’ feature dimensions, where the outputs for each model were set to one. Given that the task is binary classification, this allows for the model to produce values between 0 and 1 in order to represent a prediction for speech detection. Furthermore, we use binary cross entropy with logits loss for the student losses and binary cross-entropy for the teacher loss function to accurately predict the binary classification task and replicate the training procedure implemented in [4].

Moreover, after initial hyperparameter testing, we found that the Adam optimizer [31] was best suited for the optimization algorithm. Additionally, in each distillation experiment, we employed a learning rate of 0.001. The temperature parameter, which is used to soften the probability distribution of the logits, was set to 5. The alpha parameter, which controls the balance between the distillation loss and student loss in the total loss function, was set to 0.2. Finally, an early stopping method was used to prevent overfitting. The method involved comparing the present validation loss with the best validation loss. Furthermore, a patience parameter was introduced and set to 3 in order to ensure that if the loss failed to improve over a predetermined number of epochs specified by the patience parameter, the training of the model would be completed.

The evaluation metrics used to measure student model performance include the F1 score and the Area Under the Receiver Operating Characteristic Curve (AUC) score. The F1 score is a statistical measure used to evaluate the accuracy of a binary classifier, which can be seen as the harmonic mean of precision and recall. It provides a single performance measurement that balances both the false positives and false negatives [32]. On the other hand, the AUC score represents the likelihood that the classifier will rank a randomly chosen positive instance higher than a randomly chosen negative one. It measures the area under a curve that plots the true positive rate (TPR) against the false positive rate (FPR), offering an aggregate measure of performance across all possible classification thresholds [4]. Both the F1 score and AUC score were chosen to evaluate the student models based on the metrics employed in the training of the teacher model.

### 2.5. Software

The python programming language (3.10.11) was used throughout the study. The preprocessing pipeline was developed using EcoVAD [4], which utilizes Librosa v.0.8.1 [33] and Pydub v.0.25.1 [34] as the audio processing libraries. The data visualizations were performed using matplotlib [35]. The pandas [36] and NumPy [37] libraries were used for data loading and preprocessing. PyTorch (2.0.0) [38] was used for developing the deep learning models. The Scikit-learn [39] library was used for the evaluation of the models. The Google Colaboratory Environment [40] was used for training the models.

## 3. Results

### 3.1. Refinement of Knowledge Distillation Techniques

The performance of student models employing various knowledge distillation techniques was assessed using median Area Under the Curve (AUC) and F1 scores, providing robust central tendency measures appropriate for our data’s non-normal distribution (see Table 2). In multiple experiment runs without fixed seeds, soft target distillation yielded a median AUC of 0.98625 with a confidence interval of 0.9704 to 0.989, and a median F1 score of 0.95395 with a confidence interval of 0.9216 to 0.9596. Feature-based distillation exhibited a median AUC of 0.98795 with a confidence interval of 0.98755 to 0.99015 and a median F1 score of 0.95460 with a confidence interval of 0.95015 to 0.9583. Relational-based distillation demonstrated a median AUC of 0.98905 with a confidence interval of 0.9879 to 0.9898 and a median F1 score of 0.95900, with a confidence interval of 0.9538 to 0.96125.

A pairwise comparison of the different distillation methods, assessed by the Mann–Whitney U test, did not reveal statistically significant differences in median AUC or F1 scores between the distillation methods (all *p*-values > 0.05). This indicates that the performance of student models is consistent across different distillation methods, suggesting that while the refinement of knowledge distillation techniques did not improve the performance of the resulting models, no substantial reduction in performance was observed either.

### 3.2. Impact of Parameter Reduction and Efficiency on Model Accuracy

**Figure 1:**
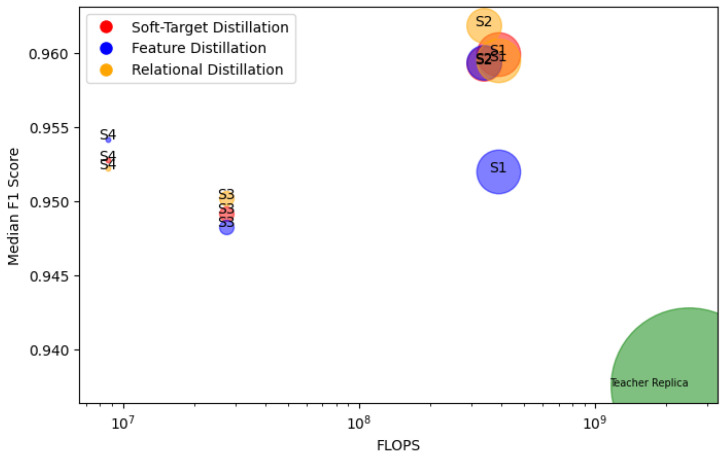
Varied distillation technique results per student with respect to FLOPS and Size. S1–S4 corresponds to the four student models, while the teacher replica is the EcoVAD model. The size of the circles corresponds to the number of parameters.

The reduction in parameters, FLOPs, multiplications, and memory utilization had varied accuracies across different distillation techniques (Figure 1). Despite these reductions, the F1 scores of the student models did not decrease when compared with the teacher-replica model. For instance, Student 1, with only 4,662,017 parameters and 388,459,000 FLOPs, achieved a median F1 score of 0.9552 in the relational distillation method, which was higher than the teacher-replica model’s F1 score of 0.9376.

The results indicate that the models are not in alignment with the assumption that a direct linear relationship exists between reductions in model parameters—inclusive of floating-point operations per second (FLOPs), multiplications, and memory utilization—and model accuracy, as Student 2 and Student 4 outperformed Student 1 and Student 3, respectively.

### 3.3. Performance of Lightweight Student Models on Playback Dataset

**Figure 2:**
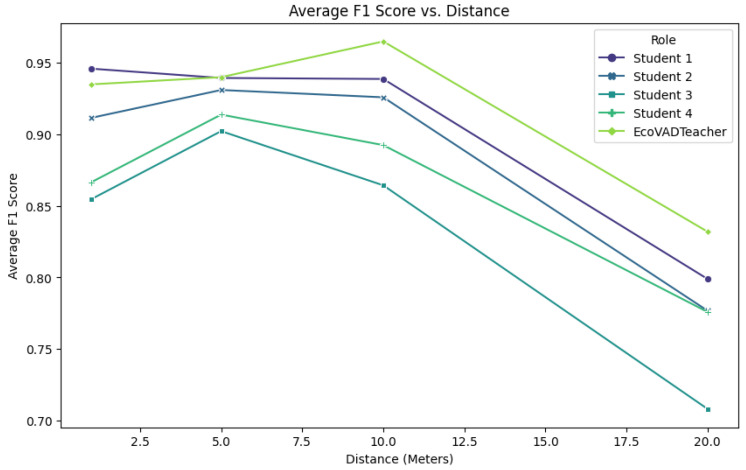
Avg. F1 scores based on distance on the playback evaluation data set for relational-based models. Please note: In this figure, we report mean rather than median scores to facilitate comparison with [4].

In terms of performance, the student models demonstrated comparable, and in one instance superior, performance relative to the EcoVAD teacher model (Figure 2). For instance, Student 1 achieved average F1 scores of 0.94595, 0.93945, 0.93875, and 0.79895 at 1, 5, 10, and 20 m, respectively, compared with the EcoVAD teacher model’s average F1 scores of 0.93500, 0.94000, 0.96500, and 0.83200 at the same distances.

Furthermore, while the avg. F1 score across all distances for the EcoVAD model was 0.917, the student averages were 0.905, 0.886, 0.832, and 0.862 for Students 1–4, respectively. These results indicate that efficient, lightweight student models can achieve comparable performance relative to the more complex EcoVAD teacher model.

These results highlight the potential of using knowledge distillation techniques for generating efficient, lightweight models for VAD tasks. Furthermore, these models maintain their accuracy despite significant reductions in parameters, FLOPs, multiplications, and memory utilization.

## 4. Discussion

The goal of this study was to build an efficient general-purpose algorithm for voice detection in environmental audio by comparing different knowledge distillation techniques and student model architectures, more specifically, using the EcoVAD model [4] as a teacher and variations in MobileNetV3-Small-Pi [18] as student models to compare knowledge distillation techniques for designing an efficient EcoVAD model. The results of this study were compared with the EcoVAD model on a playback dataset to evaluate the robustness of the efficient EcoVAD models on different landscapes with varying distances.

This study demonstrates that efficient, lightweight student models can indeed achieve comparable performance relative to the EcoVAD teacher architecture using knowledge distillation and efficient student architectures. Students 1 and 2 maintained similar avg. F1 scores on the playbacks dataset using the relational distillation models when compared with the EcoVAD teacher model. This outcome supports the findings of previous research that distillation techniques can be used to create smaller, more efficient models without a significant reduction in accuracy [11]. Furthermore, the statistical analyses conducted across various distillation techniques revealed no significant effect attributable to the refinement of knowledge distillation processes on enhancing the performance of student models. Interestingly, in both feature-based and relational distillation experiments, Student 2’s architecture outperformed Student 1’s in the VAD task on the test dataset; however, Student 1 outperformed Student 2 on the evaluation playback dataset. This could be the result of Student 2 being overfitted on the test set; however, further testing would need to be conducted in order to determine if this is the case.

Given their reduced computational demands, these student models are well-suited for deployment in edge devices, where efficiency is paramount. This study also demonstrates that reductions in parameters, FLOPs, multiplications, and memory utilization do not necessarily result in a significant decrease in model accuracy. However, the results are not linear. The student models’ performances on the evaluation dataset demonstrated that while Student 1 and Student 2 outperformed the smaller models, Student 4 consistently outperformed Student 3 on both the test set and playback dataset. This could be the result of certain architectural design features between student models, such as a difference in the expansion ratio and SE block implementation; however, further testing would need to be carried out in order to validate these claims.

## 5. Conclusions

This study demonstrates that efficient student models can achieve comparable performance to EcoVAD. The findings indicate that MobileNetV 3-Small-Pi [18] can serve as a backbone for building efficient EcoVad models capable of achieving results comparable to the EcoVAD teacher model [4]. This study suggests that Student 1 illustrates the feasibility of deploying effective lightweight EcoVAD models on small-edge devices for real-time ecological monitoring. These advancements are crucial for the field of ecological monitoring, offering a scalable solution for biodiversity assessment and the monitoring of human impacts on natural habitats.

The results of the current study are subject to certain limitations. This study incorporated a limited range of distillation techniques; therefore, other distillation methods could serve to improve upon the current results. Additionally, the experiments ran were nondeterministic; therefore, the implementation of a fixed seed could potentially enhance the reproducibility of these experiments. Moreover, portions of the data used to generate the synthetic dataset are proprietary and therefore restricted to research purposes only. Future research could explore the use of other distillation techniques while further investigating different variations in the student EcoVAD models presented. Additionally, research could investigate the performance of these models in real-world settings.

Our work contributes to ongoing efforts to expand eco-acoustic monitoring technologies. Our focus on efficiency and the deployment feasibility of VAD models paves the way for such algorithms to be deployed on small embedded devices, such as Raspberry Pi, to detect and remove human voices where privacy is a strong constraint or equally well to monitor patterns of human disturbance. The present study indicates that the optimization and design of efficient lightweight student models can lead to results comparable to the larger EcoVAD model. While the current study is in no way a thorough investigation into efficient VAD model design, it can be considered a contribution toward the design of an efficient general-purpose algorithm for voice detection in ecological settings.

## Figures and Tables

**Table 1:** Summary of different teacher and student model characteristics. Avg. inference time is defined as the average time taken by the model to make a prediction on a single input instance, measured over 100 trials.

Model	Parameters	Layers	FLOPS	Multiplications	Memory (MB)	Avg. Inference Time (s)
Teacher	59,568,769	20	2,485,390,000	1,242,700,000	227	0.17
Student 1	4,662,017	215	388,459,000	194,230,000	17	0.038
Student 2	2,930,177	179	337,257,000	168,628,000	11	0.042
Student 3	502,793	179	27,353,400	13,676,700	1.91	0.0087
Student 4	52,253	114	8,648,350	4,324,170	0.19	0.0050

**Table 2:** Table of results for different student models employing distinct distillation techniques. We report median AUC and F1 scores of the student models and distillation methods with bootstrap confidence intervals given in brackets.

Model	Soft Target Distillation	Feature-Based Distillation	Relational-Based Distillation
Student 1	AUC: 0.9892	AUC: 0.9880	AUC: 0.9899
	F1: 0.9599	F1: 0.9520	F1: 0.9595
Student 2	AUC: 0.9908	AUC: 0.9899	AUC: 0.9897
	F1: 0.9593	F1: 0.9594	F1: 0.9619
Student 3	AUC: 0.9850	AUC: 0.9874	AUC: 0.9880
	F1: 0.9492	F1: 0.9483	F1: 0.9502
Student 4	AUC: 0.9870	AUC: 0.9877	AUC: 0.9878
	F1: 0.9528	F1: 0.9542	F1: 0.9552
Overall	AUC: 0.98625 [0.9704–0.9895]	AUC: 0.98795 [0.98755–0.99015]	AUC: 0.98905 [0.9879–0.9898]
	F1: 0.95395 [0.9216–0.9596]	F1: 0.95460 [0.95015–0.9583]	F1: 0.95900 [0.9538–0.96125]

## Data Availability

The data presented in this study are available on request from [4].

## Abbreviations

The following abbreviations are used in this manuscript:

CNN	Convolutional Neural Networks
AST	Audio Spectrogram Transformer
DNN	Deep neural network
